# Skin Equivalent Tissue-Engineered Construct: Co-Cultured Fibroblasts/ Keratinocytes on 3D Matrices of Sericin Hope Cocoons

**DOI:** 10.1371/journal.pone.0074779

**Published:** 2013-09-13

**Authors:** Sunita Nayak, Sancharika Dey, Subhas C. Kundu

**Affiliations:** Department of Biotechnology, Indian Institute of Technology, Kharagpur, India; Institute for Frontier Medical Sciences, Kyoto University, Japan

## Abstract

The development of effective and alternative tissue-engineered skin replacements to autografts, allografts and xenografts has became a clinical requirement due to the problems related to source of donor tissue and the perceived risk of disease transmission. In the present study 3D tissue engineered construct of sericin is developed using co-culture of keratinocytes on the upper surface of the fabricated matrices and with fibroblasts on lower surface. Sericin is obtained from “Sericin Hope” silkworm of *Bombyx mori* mutant and is extracted from cocoons by autoclave. Porous sericin matrices are prepared by freeze dried method using genipin as crosslinker. The matrices are characterized biochemically and biophysically. The cell proliferation and viability of co-cultured fibroblasts and keratinocytes on matrices for at least 28 days are observed by live/dead assay, Alamar blue assay, and by dual fluorescent staining. The growth of the fibroblasts and keratinocytes in co-culture is correlated with the expression level of TGF-β, b-FGF and IL-8 in the cultured supernatants by enzyme-linked immunosorbent assay. The histological analysis further demonstrates a multi-layered stratified epidermal layer of uninhibited keratinocytes in co-cultured constructs. Presence of involucrin, collagen IV and the fibroblast surface protein in immuno-histochemical stained sections of co-cultured matrices indicates the significance of paracrine signaling between keratinocytes and fibroblasts in the expression of extracellular matrix protein for dermal repair. No significant amount of pro inflammatory cytokines (TNF-α, IL-1β and nitric oxide) production are evidenced when macrophages grown on the sericin matrices. The results all together depict the potentiality of sericin 3D matrices as skin equivalent tissue engineered construct in wound repair.

## Introduction

Skin basically consists of two layers, outer epidermis and inner dermis. The epidermis is rich with layers of keratinocytes which forms sub layers of different stages of keratinocytes upon the basement membrane. The basal keratinocytes apposed to the basement membrane, synthesizes its own basement membrane including laminin isoforms, type IV collagen and anchoring fibril. During re-epithelialization the keratinocyte makes contact with basement membrane collagens (type IV and VII) [1]. Moreover type IV collagen promotes *in vitro* migration of human keratinocytes [2]. The dermis chiefly consists of fibroblast and extra cellular matrix (ECM) of mainly type I collagen. In skin wounds where both these layers (epidermis and dermis) are lost, prime focus is to replace both the layers for effective healing. Plenty of different dressings and techniques are available for managing certain types of open wounds. An ideal wound dressing should be easy to apply, capable of promoting moist wound healing, be non-toxic, non-allergenic, thermally insulating, impermeable to bacteria, allows proper gaseous exchange and cost effective [3]. Traditional dressings such as gauze, paraffin gauze and biological dressings etc are being replaced by various synthetic materials due to certain disadvantages [4]. However issues related to integration of synthetic materials into the body remains uncertain. The uses of autograft, allografts or xenografts are alternative approach for skin repair. Although the expensive transplantation procedures, risk to infection, insufficient donor availability, risk of disease transmission and immune rejection make them less suitable [5,6].

The current approach involves the development of tissue engineered constructs of biomaterials using patients own cells for skin replacement. This system avoids immunological rejection and improves material integration into the body. The three-dimensional structure of biomaterial matrices provides the micro-environment experienced by the cells in the living organism allowing intercellular interactions with more realistic, biochemical and physiological manner. Moreover to various intrinsic and extrinsic stimuli such as to temperature, pH, nutrient absorption, transport and differentiation, cells react and behave more like as *in vivo* conditions [7]. Recently co-cultured grafts comprising of dermal and epidermal modules capable of autologous skin replacement are of prime interest due to their accelerated healing processes [8]. Skin wound healing basically comprises epidermal tissue regeneration regulated by interaction of cytokines and growth factors with controlled functional behaviour of keratinocytes and dermal fibroblasts [9-11]. Keratinocytes proliferation in co-culture model is found to be based on active interplay between both cell types in a double paracrine mechanism, cell-matrix interactions and direct cell-cell contact signals [9,12,13]. Moreover, increased expression of several growth factors and interleukins such as IL-1, IL-6, IL-8, granulocyte macrophage colony stimulating factor (GM-CSF), transforming growth factor (TGF) alpha and beta, NGF, PDGF as well as several members of the FGF family are reported in skin as well as in keratinocytes and fibroblasts culture [9]. This paracrine and autocrine growth factor regulation in co-culture skin draft indicates their functional significance in the dermal-epidermal interactions that regulates tissue repair and homeostasis [14].

A variety of biopolymer like agarose, alginate, carageenan, gellan, chitosan, dextran, collagen, gelatin and hyaluron are considered to be suitable biomaterial based wound dressing materials. Silk sericin is a family of adhesive silk protein synthesized in middle silk glands of silkworms [15]. It envelops fibroin fibers in cocoon [16]. Sericin usually constitutes 20–30% of silk protein in cocoon [17,18]. It consists of amino acids most of which have strong polar side groups such as hydroxyl, carboxyl, and amino groups with high serine content contributing to its high hydrophilicity [19]. Sericin is non toxic, antioxidant agent, with antibacterial, UV resistant and anti apoptotic properties [17]. Other biological activities it comprises are anti tyrosinase, anticoagulation and anti-cancerous activities [17,20]. Additionally, it supports cell growth and differentiation and has been considered to act as cell culture supplement in serum free media [21]. Sericin as biomaterial in several different forms like cross-linked films, gels, sponges of sericin/gelatin and sericin/polyvinyl alcohol (PVA), and electrospun materials are reported [20,22,23]. Sericin is treated as a by-product, produced during the processing of raw silk fibres in industries. It is removed from the silk fibres by the different procedures specifically like high alkali, high-pressure steam heating, boiling, and enzymatic treatment [20,24]. A silkworm race known as ‘Sericin Hope’ is developed that secretes sericin in large quantity as high as 98.5% at the cocoon stage. This new silkworm race is developed by cross breeding an Nd mutant (naked pupa) and a high cocoon yielding strain KCS83 [25]. The sericin from “Sericin Hope” cocoon, named “Virgin Sericin” can be obtained by autoclaving at 110 °C for 10 min in water with less hydrolysis [25]. This paper deals with the fabrication of 3D sericin construct, co-cultured with keratinocytes and fibroblasts cell to modulate wound healing *in vitro*. Co-culture on sericin matrices by layering two cell types one on top of the other is carried out in this study. The interactions between keratinocytes and fibroblasts and their paracrine interaction *in vitro* in the sericin construct are investigated. We examine various markers and cytokines expressed by the co-cultured sericin 3D functional matrices and its ability to reconstruct an epidermal layer as biological proof to serve as a potential skin equivalent.

## Results

### Preparation of porous matrices

**Figure 1:**
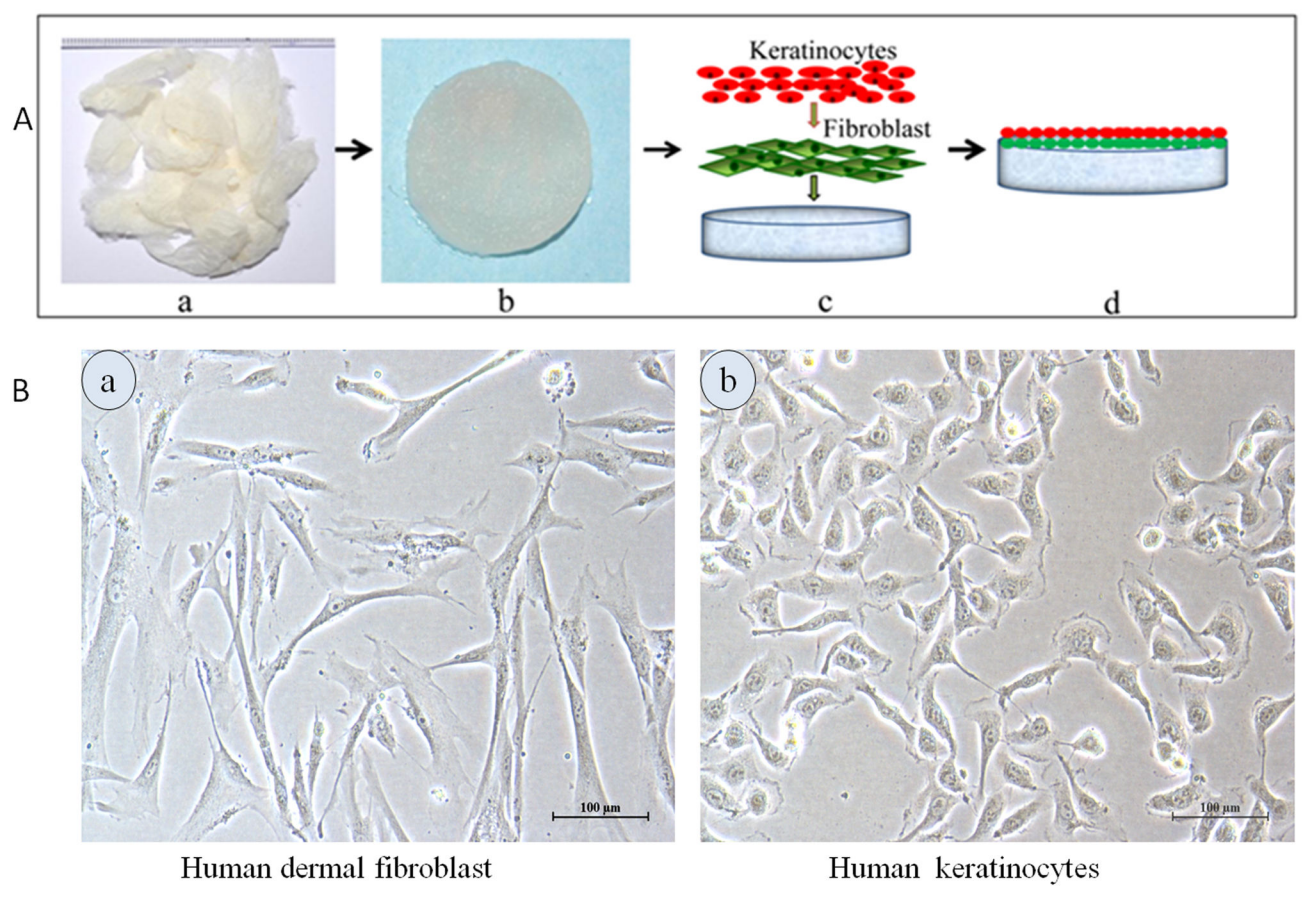
The schematic representation showing fabrication of sericin matrices from “Sericin hope” silkworm cocoons and co-culture system (A). Keratinocytes-fibroblast coculture was done by seeding fibroblasts on sericin matrices pre-soaked in DMEM medium at day 0. On day 2, keratinocytes were directly seeded on the surface of sericin matrices containing fibroblasts. The cells attached to the surface of matrices and proliferated over time to cover the surface of the entire sericin matrices. Optical microscope images (B); of (a) human dermal fibroblasts and, (b) human keratinocytes cells used in the experiments. Scale bars = 100 micrometer.

Porous sericin matrices via freeze dried method, crosslinked with genipin (0.5% w/v) are obtained. The schematic representation of preparation of porous sericin matrices and further co-culture with fibroblasts and keratinocytes is shown in Figure 1A. Human dermal fibroblasts and human keratinocytes used for seeding the matrices are shown in Figure 1B (a) and Figure 1B (b) respectively.

### Scanning Electron Microscopy (SEM) Analysis

**Figure 2:**
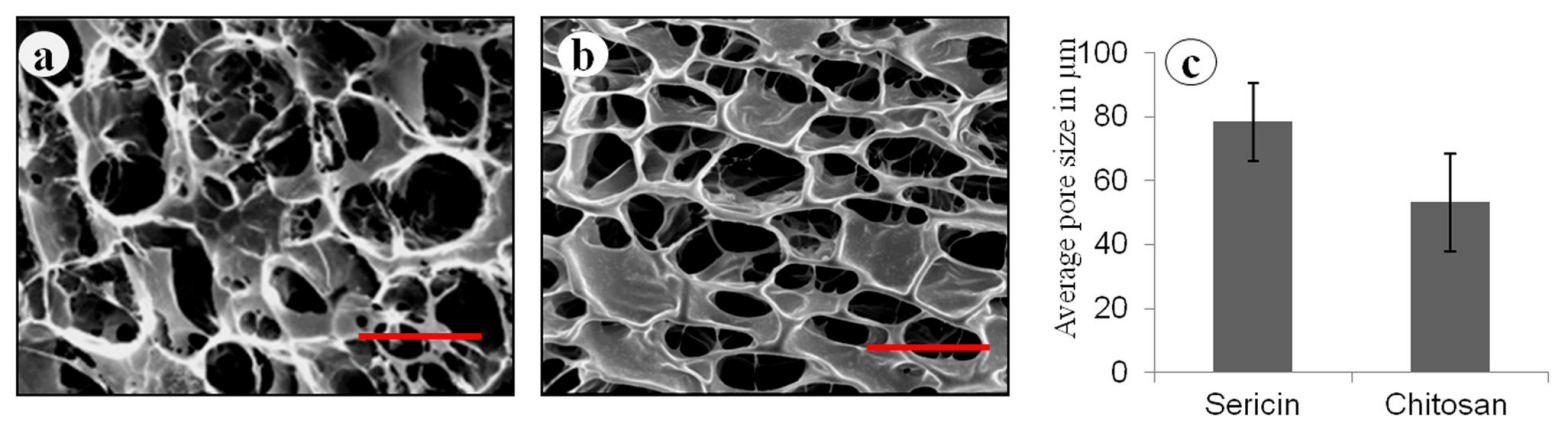
Scanning electron micrograph of surface of 2% (a) sericin, (b) chitosan matrices and (c) average pore size of sericin and chitosan matrices. The magnification bar represents 100 micrometer.

Porous sericin and chitosan matrices fabricated are evaluated by SEM as shown in Figure 2. The pore diameters of the matrices are estimated to be in the range of 98–51 µm and 87–31 µm for sericin (Figure 2a) and chitosan (Figure 2b) matrices respectively. The mean diameter of 78 ± 12 µm, and 53 ± 15 for sericin and chitosan respectively is measured by using image analysis software (ImageJ) (Figure 2c).

### Fourier transform infrared spectroscopy (FTIR) measurement

**Figure 3:**
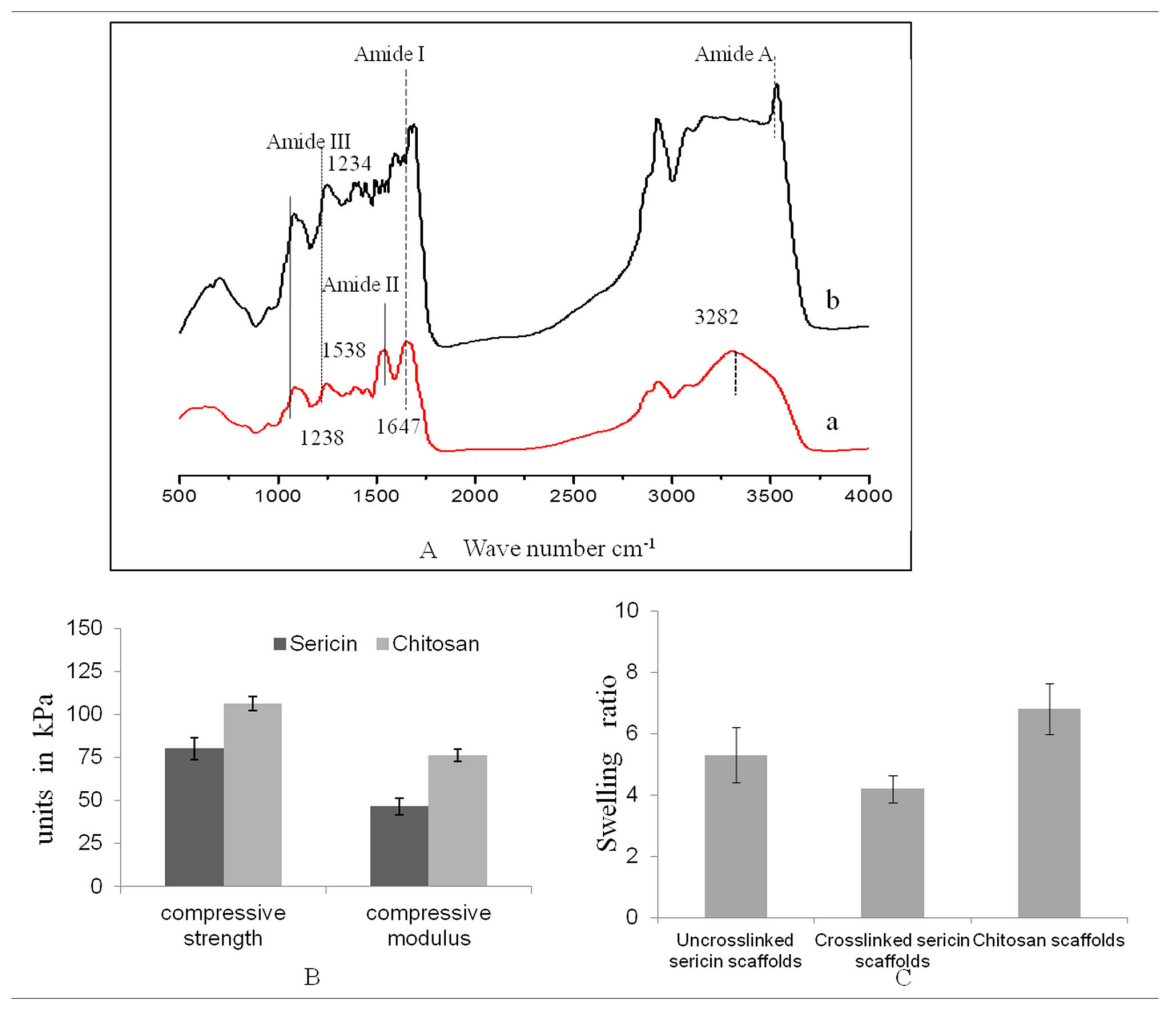
FTIR spectra of sericin porous matrices (A). Non-crosslinked (a) and genipin (b) crosslinked sericin matrices; Mechanical properties (B); and swelling ratio (C) of sericin and chitosan porous matrices in water at 37 °C. Error bars represent the standard error of the mean (n=3).

FTIR spectroscopy indicates typical absorption bands sensitive to molecular conformation of proteins therefore a significant tool to analyze protein structure at the molecular level. In order to determine hope sericin structural modifications due to the crosslinking process, FTIR was carried out of sericin and genipin crosslinked sericin matrices (Figure 3A). The FTIR spectrum shows several absorption bands corresponding to amide A, I, II and III. The amide A band absorption at ~3500 cm^-1^ is associated with the N–H stretching mode. Amide I is most powerful for estimating protein secondary structure arising from the carbonyl C=O double stretching mode in region from between 1600 and 1700 cm^-1^ and amide II has been ascribed to the deformation of N–H bonds and C–H stretching absorption band at 510 and 1580 cm^-1^ region. Amides III, V are very complex bands dependent on the details of the force field, the nature of side chains and hydrogen bonding. The FTIR spectrum of uncrosslinked sericin shows the characteristic bands of amide I, amide II and of amide III, respectively at 1647 cm^-1^, 1538 cm^-1^ and at 1238 cm^-1^, caused by C–N stretching vibrations, amide N-H in-plane bending vibrations and CH2 vibrations. Bands due to N–H and O–H stretching vibrations overlapped in the adsorption are seen at peak value of 3282 cm^-1^. The FTIR spectra of uncrosslinked sericin, peak at 1647 cm^-1^ assigned to the random coil conformation. However crosslinked sericin does not displays significant structural differences but exhibits relatively high intensity for the 1234 cm^-1^ band in the amide III region. More precisely, FTIR spectra of crosslinked sericin sample displays a slight increase of the intensities of the adsorption bands at 1630–1640 cm^-1^ (amide C=O stretching vibrations) and at 1540–1550 cm^-1^ (amine and amide N-H bending vibrations).

### Mechanical properties

The compressive strength and modulus of sericin and chitosan matrices are shown in Figure 3B. The compressive strength and modulus of chitosan is relatively higher than sericin matrices. In this experiment matrices were compressed to 80%. The average compressive strength of sericin and chitosan matrices is 80 ± 6 kPa and 106 ± 4 kPa respectively. Similar trend is observed for compressive modulus also. The average compressive modulus of sericin and chitosan matrices is 46 ± 4 kPa and 76 ± 3 kPa respectively.

### Swelling behavior

The swelling ratio of sericin and chitosan matrices is shown in Figure 3C. Upon immersion in water, all matrices imbibed water rapidly and reached equilibrium within 24 h. It can be seen from the (Figure 3C) that the water-retention ability of chitosan is more than crosslinked sericin matrices. The uncrosslinked matrices of sericin have a higher swelling ratio as compared to the corresponding crosslinked ones. Uncrosslinked and crosslinked sericin matrices showed SR of 5.3 and 4.2 respectively. At the same time chitosan matrices showed SR of 6.8 (*P<0.05).

### Integral stability evaluation by *in vitro* degradation

**Figure 4:**
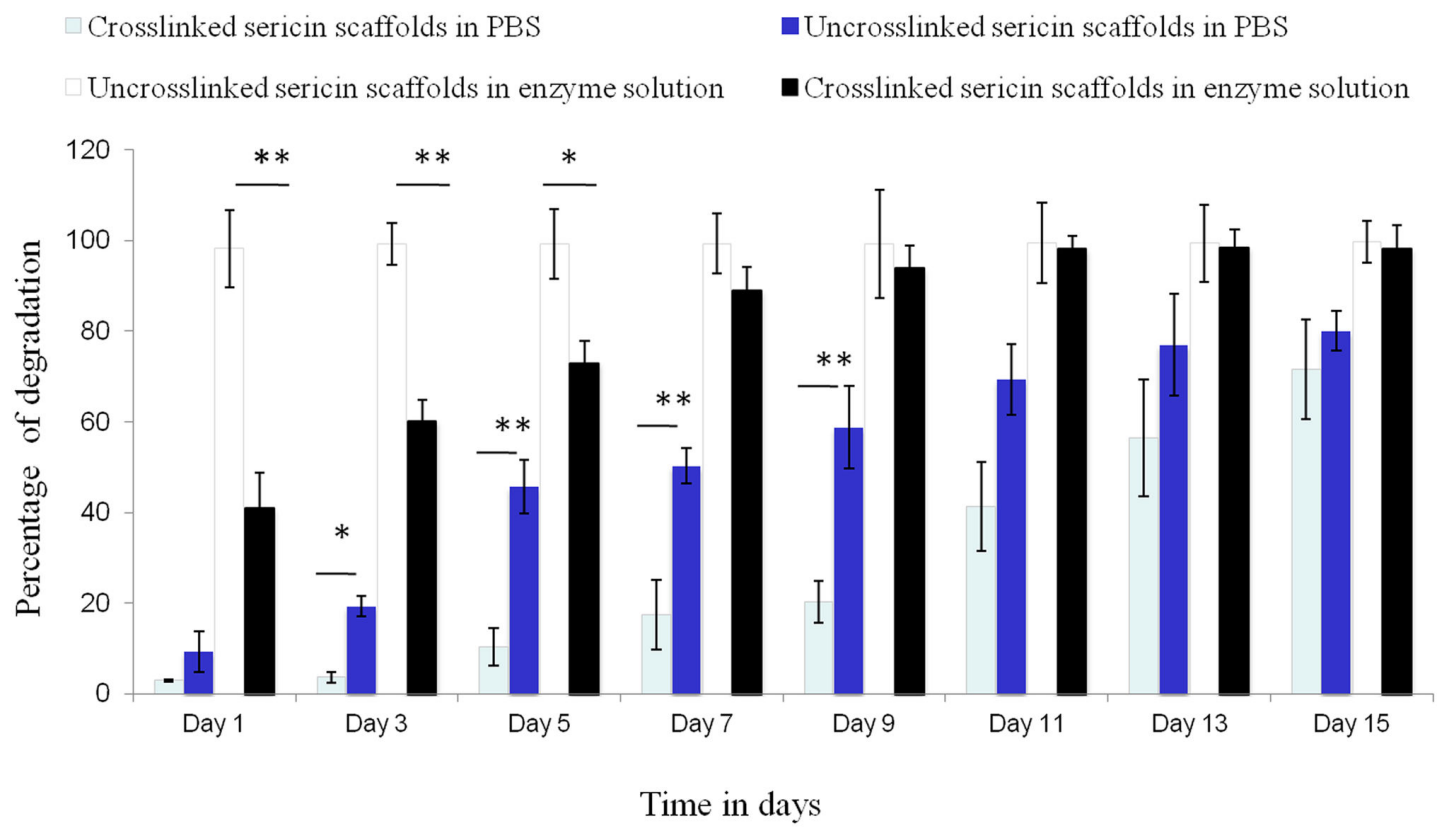
Percentage degradation in terms of weight loss of sericin crosslinked and uncrosslinked matrices in phosphate buffer saline pH 7.4 solution and in lysozyme solution at 37 °C (n= 3, Mean ±standard deviation, *p< 0.05).

Degradative changes in the form of weight loss of sericin matrices after immersion in PBS (pH 7.4) and in enzymes over specified period of 14 days is shown in Figure 4. The total weight loss for the crosslinked sericin matrices accounted for approximately 56% in day 14. While in non crosslinked sericin 80% weight loss is observed in PBS. The *in vitro* enzymatic degradation profiles of uncrosslinked and crosslinked treatment are also shown in Figure 4. The uncrosslinked sericin matrices degraded completely within 24 h. There is only ~43% degradation in the crosslinked sericin matrices within 24 h and around day 7, 89% degradation is seen. The result indicates that after performing the cross-linking treatment with 0.5% w/v genipin solution for 24 h, the anti-degradation ability of sericin matrices enhanced.

### Alamar blue assay and live/ dead staining

**Figure 5:**
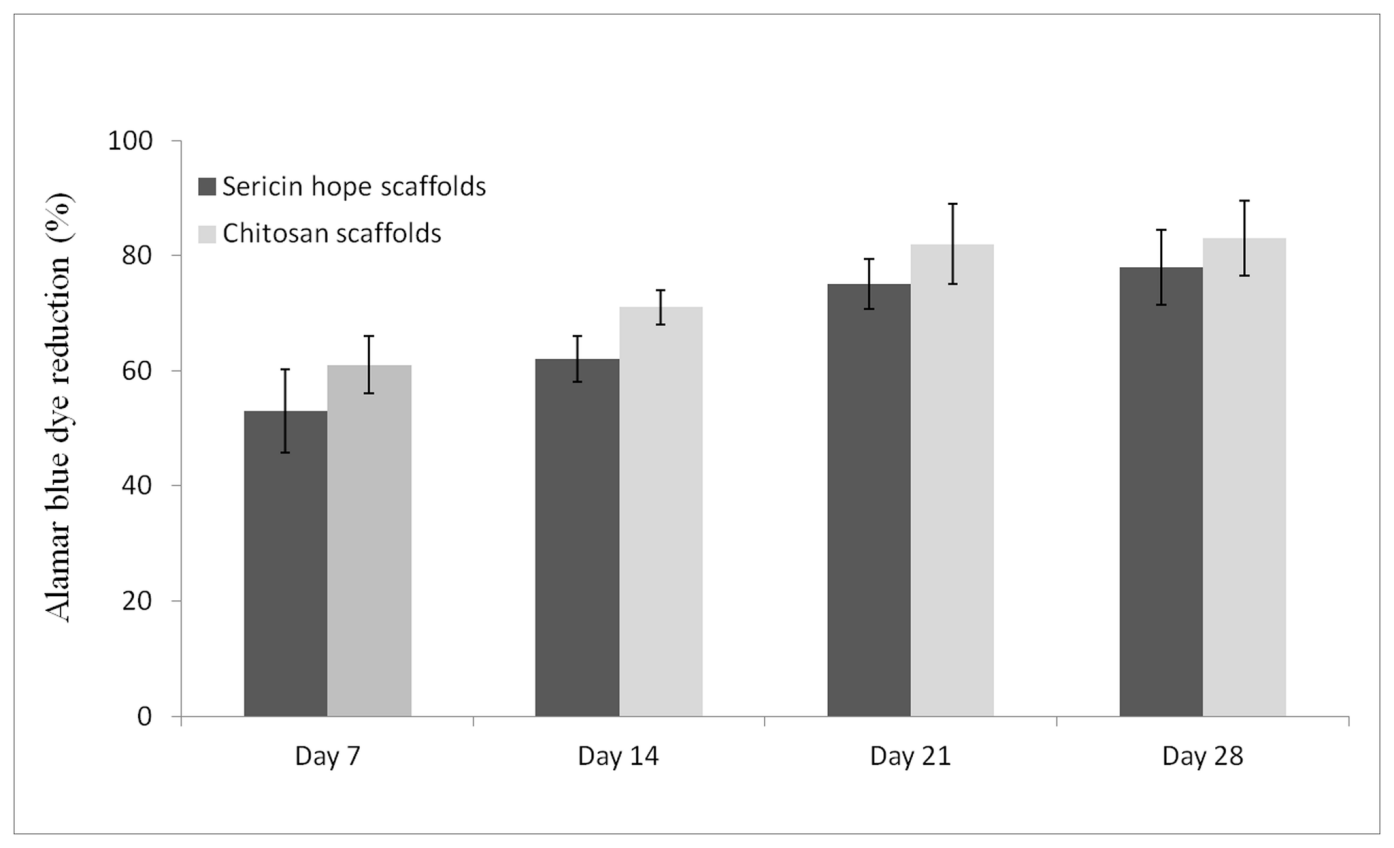
Alamar blue assay of co-cultured cells on sericin and chitosan porous matrices (n= 3, Mean ±standard deviation).

**Figure 6:**
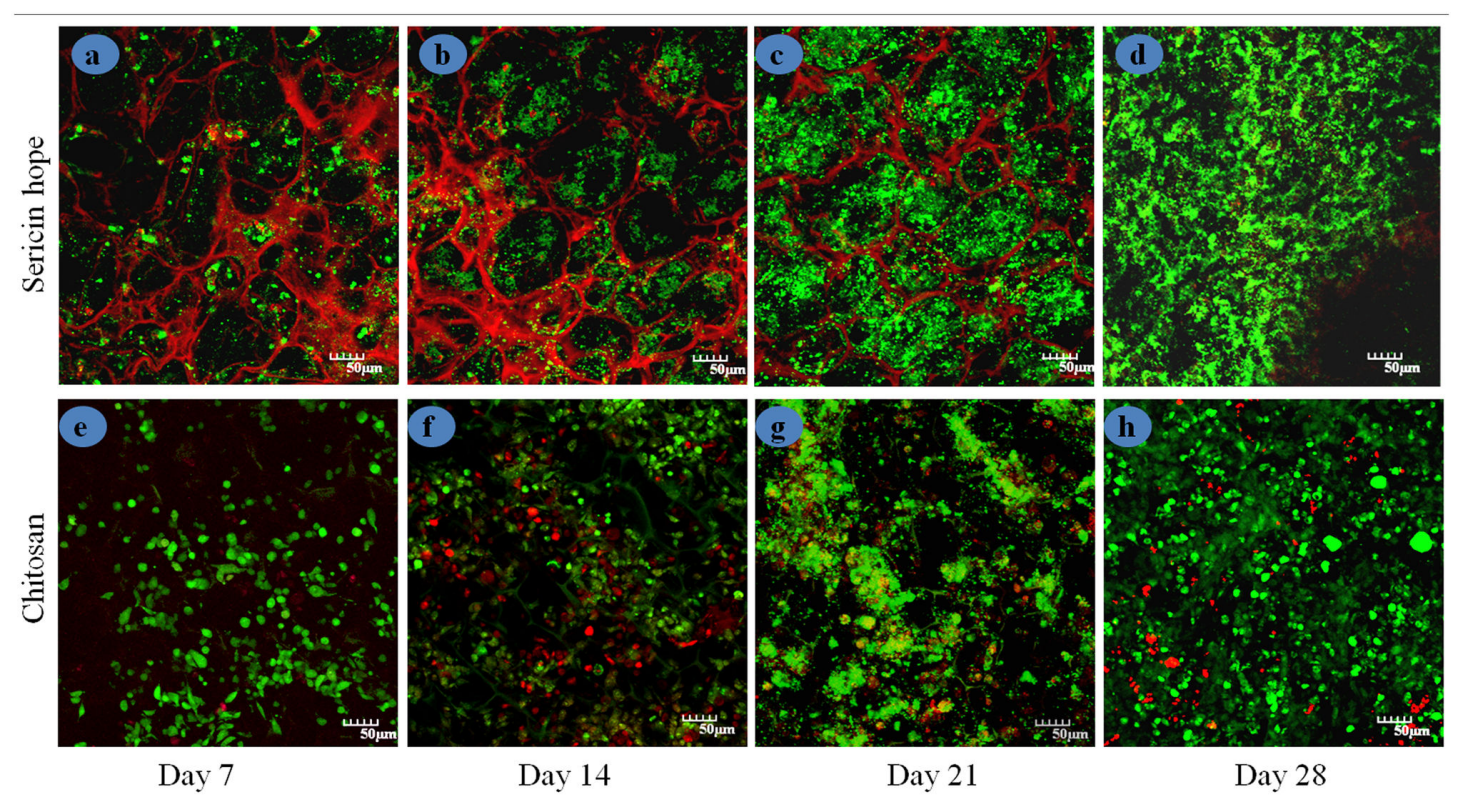
The viability of co cultured cells on sericin and chitosan porous matrices using Live/Dead assay with confocal microscopy of day 7, 14, 21 and 28 culture. Live cells produced green fluorescence and dead cells showed red fluorescence. Scale bars are 50 micrometer.

Alamar blue assay was performed to assess the cell proliferation on the sericin matrices at different time periods and is shown in Figure 5. Alamar blue is safe, non-toxic aqueous dye that is used to evaluate cell viability and cell proliferation. The Figure 5 shows that the sericin matrices in co-culture displays low cytotoxicity and high proliferative response (cell viability >78%) up to 28 days from the initial day of cell seeding. At the first day of co-culture a reduction of 53% in sericin and 61% in chitosan matrices is seen which is comparable with sericin matrices. After 2 weeks, Alamar blue reduction is further increased by 62% and 71% respectively for sericin and chitosan matrices. However in 3rd and 4th week the proliferation rate did not increased significantly in both sericin and chitosan matrices. On 3rd and 4th week Alamar reduction for hope sericin matrices are found to be 75% and 78% respectively. While in chitosan matrices the reduction are 82% and 83% respectively. Live/ dead staining was used to assess the long-term effect of sericin matrices on cell viability. Live/dead staining of co-culture matrices with fibroblasts and keratinocytes were performed at 7, 14, 21, 28 days. The cells are seen to be homogeneously distributed throughout the matrices as shown in Figure 6. The presence of mainly green and very few red cells illustrates that cell viability is maintained throughout the matrices for at least 28 days during *in vitro* co-culture.

### The growth of keratinocytes and fibroblasts in co-culture

**Figure 7:**
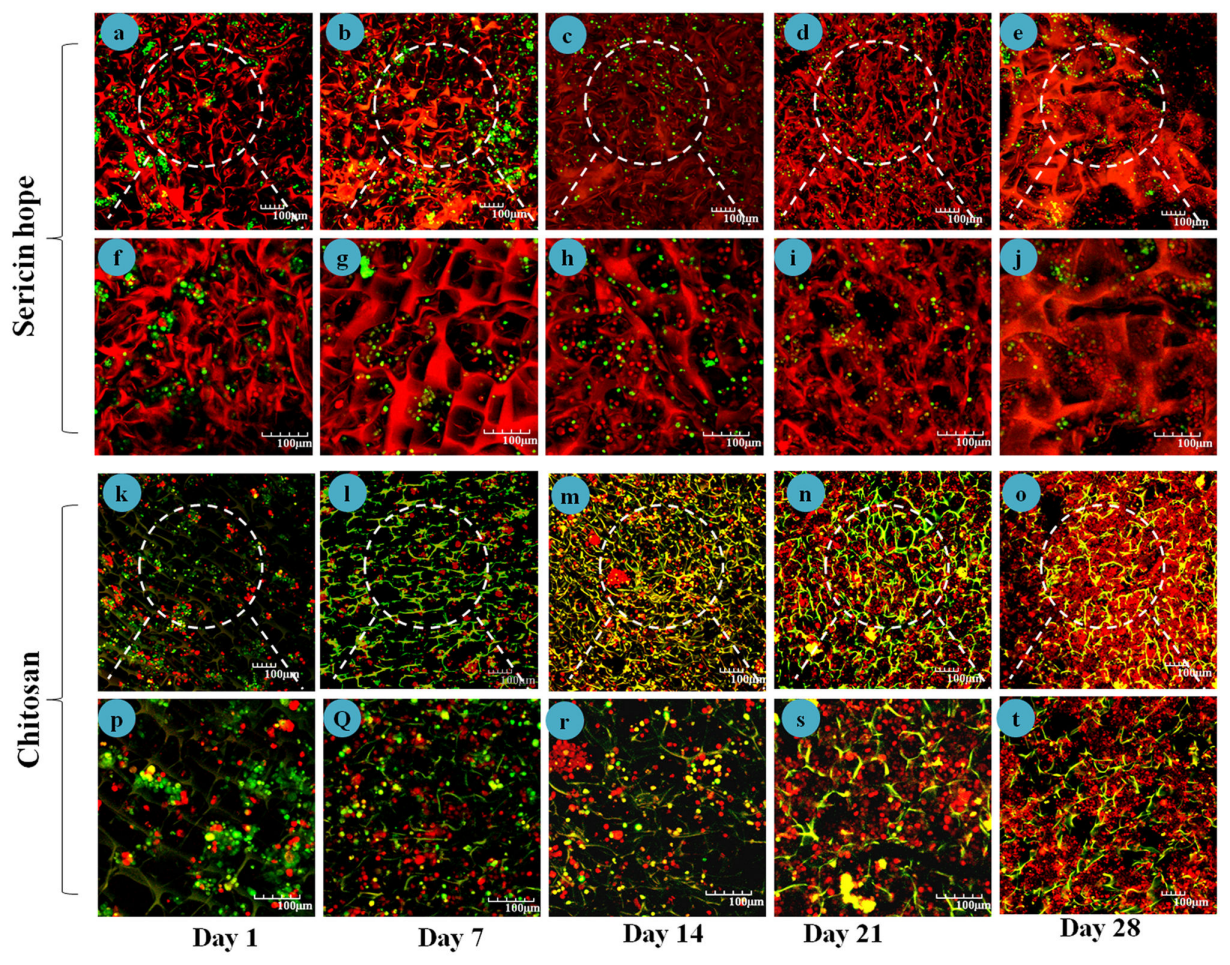
Growth and proliferation of human keratinocytes and fibroblasts on sericin and chitosan matrices observed under confocal micrographs for day 1, 7, 14, 21 and 28 days. Scale bars = 100 micrometer. Chitosan and sericin matrices show red and green auto fluorescence respectively in lasers used in confocal microscope.

**Figure 8:**
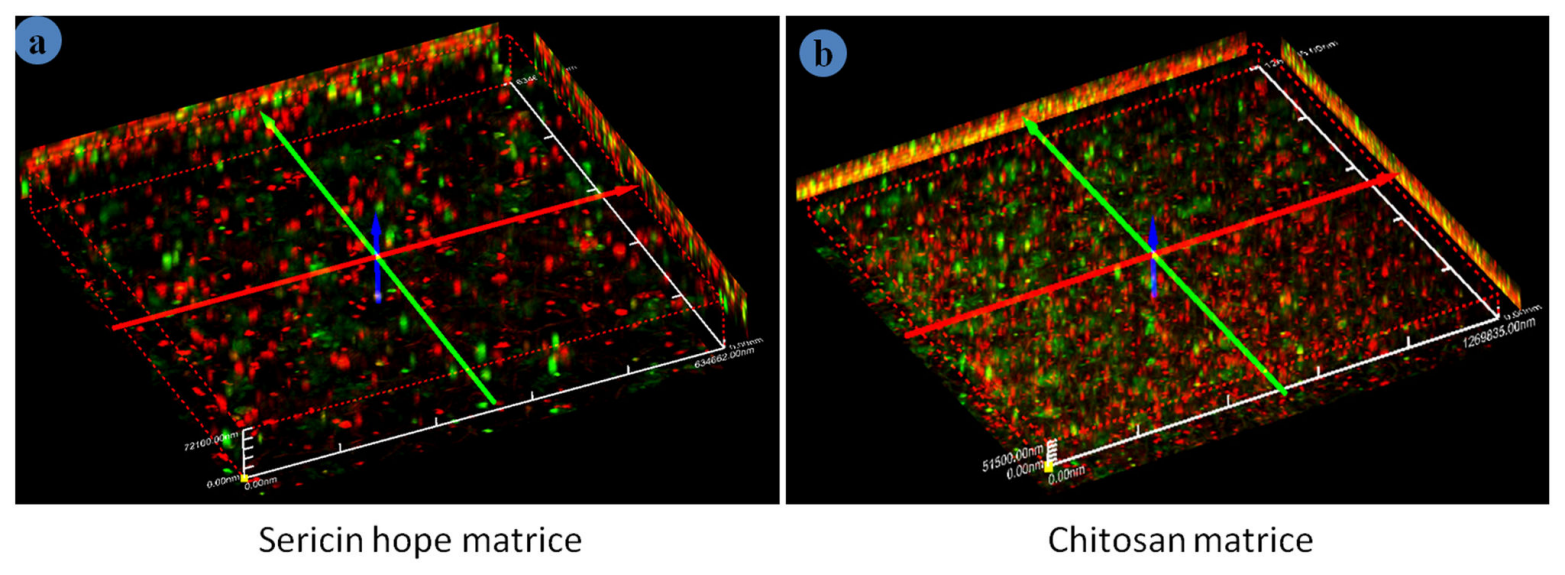
Three dimensional confocal images of fibroblasts and keratinocytes co-cultured on (a) sericin and (b) chitosan matrices showing two different cell types distribution over the matrices at day 14.

**Figure 9:**
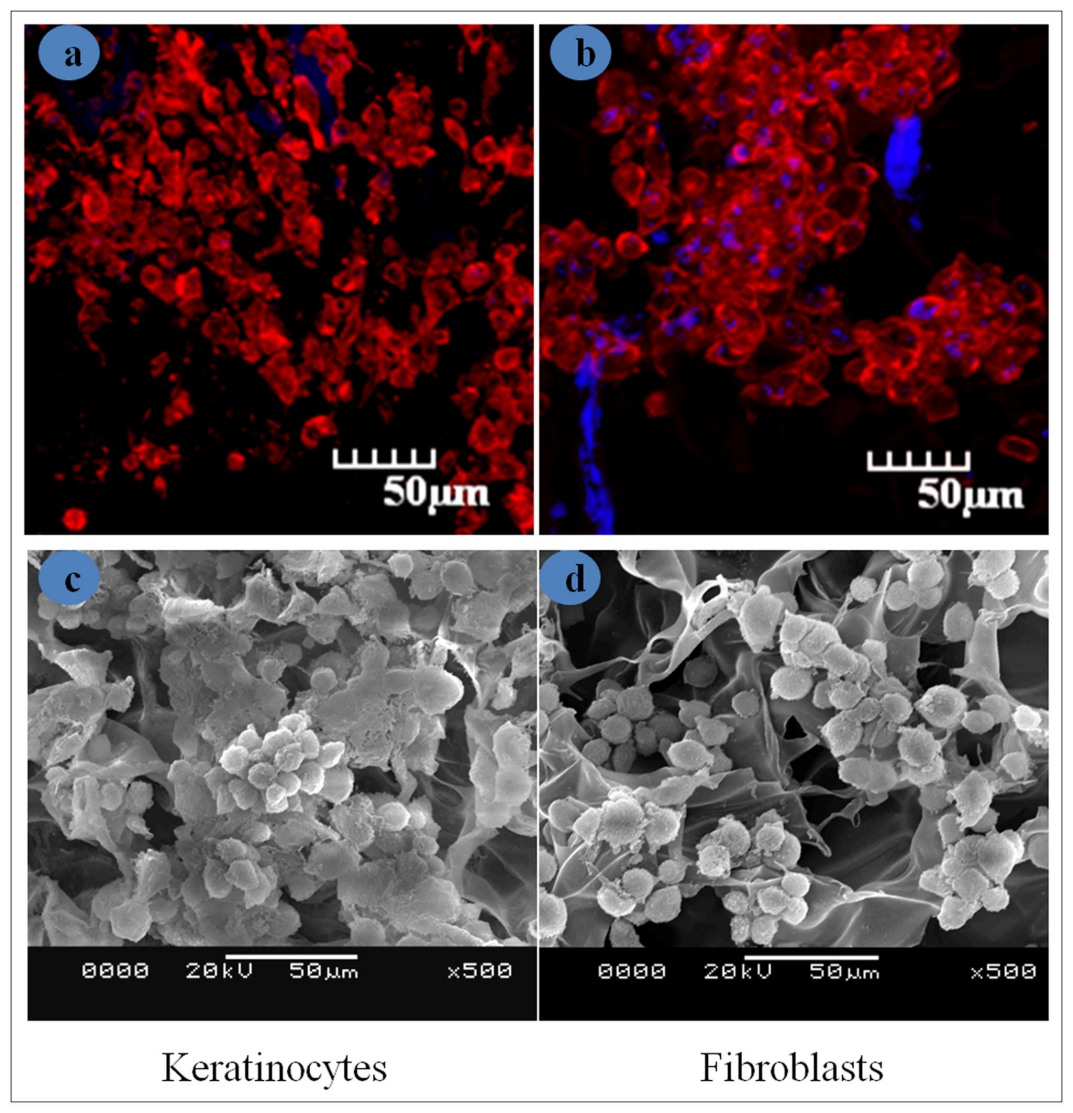
Figure represents morphology of seeded cells observed by confocal microscope and SEM on sericin matrices (a; c) keratinocytes, (b; d) fibroblasts.

To distinguish keratinocytes from fibroblasts in the co-culture, keratinocytes were stained red and fibroblasts were stained green. Seeding of cells on both sericin and chitosan matrices were kept same. Initial attachment of cells on chitosan (Figure 7 (k:p and i:q)) is more than sericin matrices as seen from day 1 (Figure 7a:f) and day 7 (Figure 7b:g) post seeding of cells. On day 14 fibroblasts overgrew, though keratinocytes are highly populated than fibroblasts and covers fibroblasts to form skin-like structures on sericin matrices (Figure 7c:h) comparable to that of chitosan matrices (Figure 7m:r). At 28 days of culture condition keratinocytes covers most of the fibroblasts (Figure 7e:j) and (Figure 7o:t) in sericin and chitosan matrices respectively. Figure 8a and b shows the homogeneity of the cell distribution of fibroblasts (green) and keratinocytes (red) throughout the sericin and chitosan matrices respectively. The adhesion and proliferation of keratinocytes and fibroblast cells are studied by seeding the cells on sericin matrices in a monoculture. The keratinocytes and fibroblasts on sericin matrices appear to have rounded morphology. Figure 9a and Figure 9c are the respective confocal and SEM images of keratinocytes grown on sericin matrices. While Figure 9b and Figure 9d are confocal and SEM images of fibroblasts grown on sericin matrices.

### TGF-β, b-FGF and IL-8 quantification by ELISA

**Figure 10:**
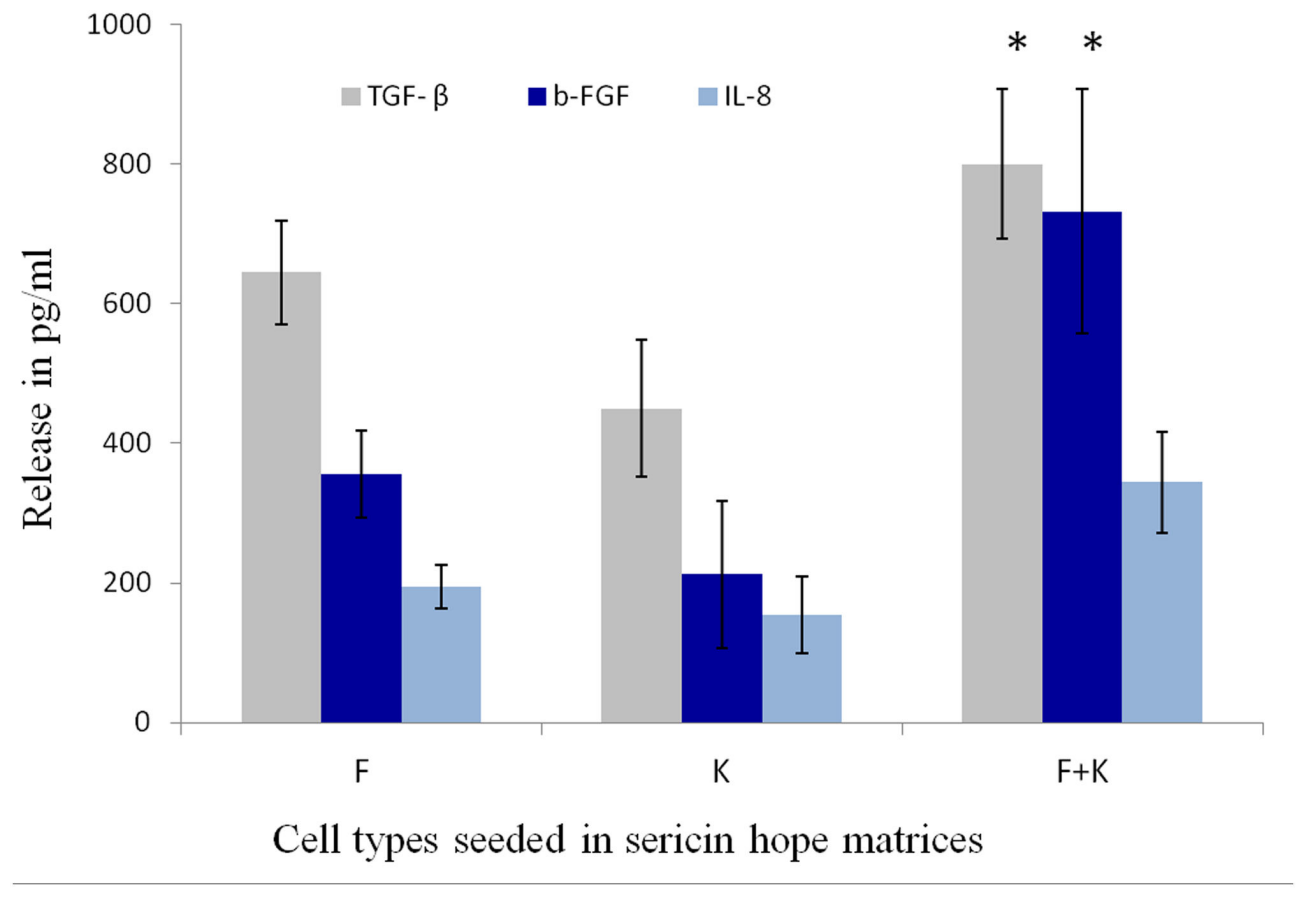
The *in vitro* release profile of the growth factors TGF beta, b-FGF and cytokine IL-8 by cocultured cells, fibroblasts and keratinocytes monoculture cells seeded on sericin matrices for 72 h, essential for epidermal morphogenesis.

Quantitative expression TGF-β, b-FGF, and IL-8 over culture time period of 72 h are shown in Figure 10. Fibroblasts, co-cultured with keratinocytes, expressed higher levels of TGF-β compared with control fibroblasts or keratinocytes in mono culture by means of ELISA. Similarly, an increased level for b-FGF is observed in keratinocytes-fibroblasts coculture. However no significant difference in expression of IL-8 was seen in the co-culture.

### H: & E staining

**Figure 11:**
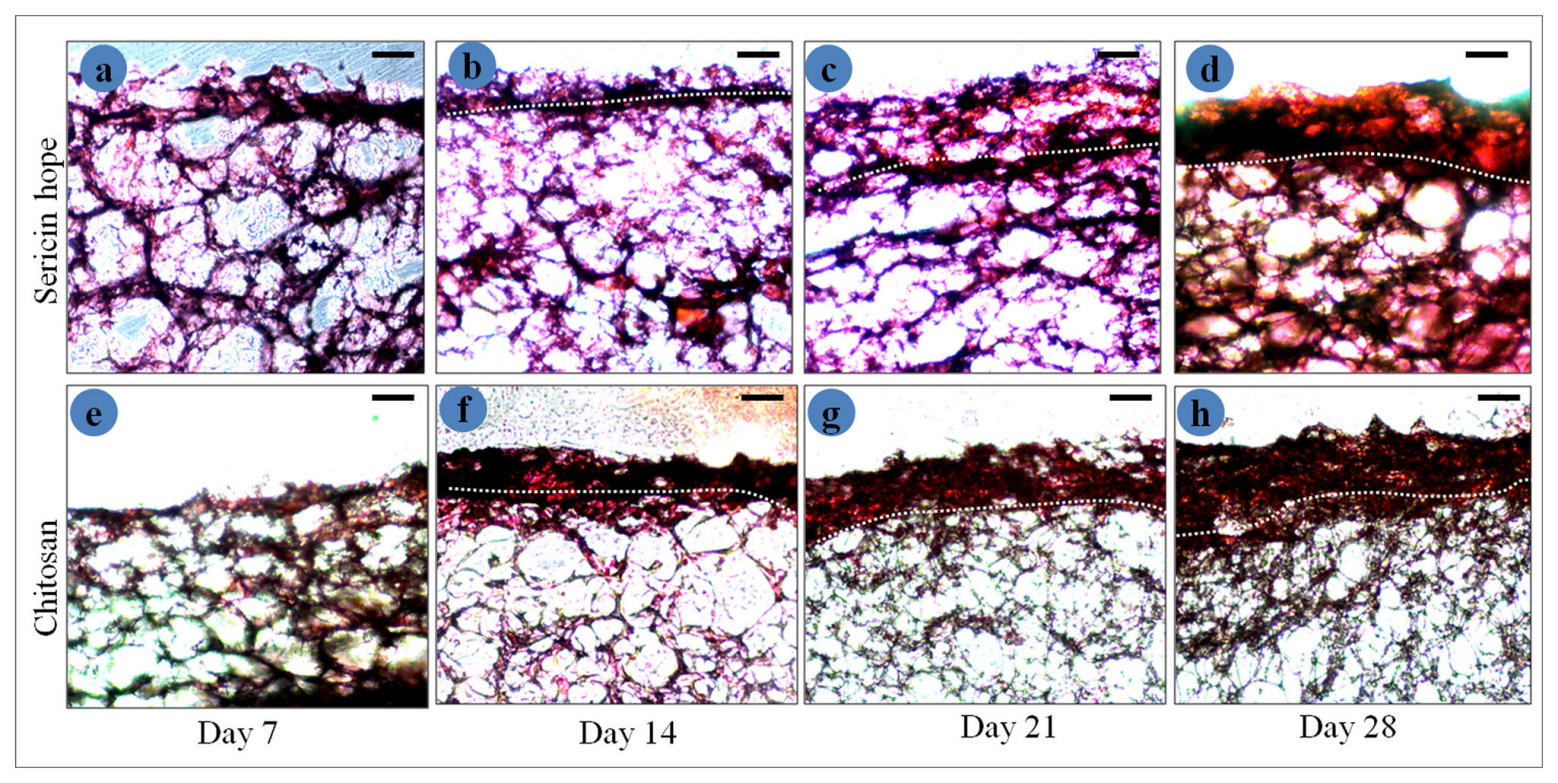
H & E stained cross-sections of co-culture constructs composed of epidermal and dermal layers formed by culturing fibroblasts and keratinocytes on the constructs for 7, 14, 21 and 28 days. White broken line outlines the border between epidermis and dermis (scale bar =100 micrometer).

Histological evaluation with H&E staining illustrates stratified epidermal layers of keratinocytes formed after 14 days of growth. H&E stained cross-sections of sericin and chitosan constructs composed of epidermal and dermal layers formed by culturing fibroblasts and keratinocytes on constructs for 7, 14, 21 and 28 days are shown in Figure 11.

### Immunohistochemical staining of co-cultured construct

**Figure 12:**
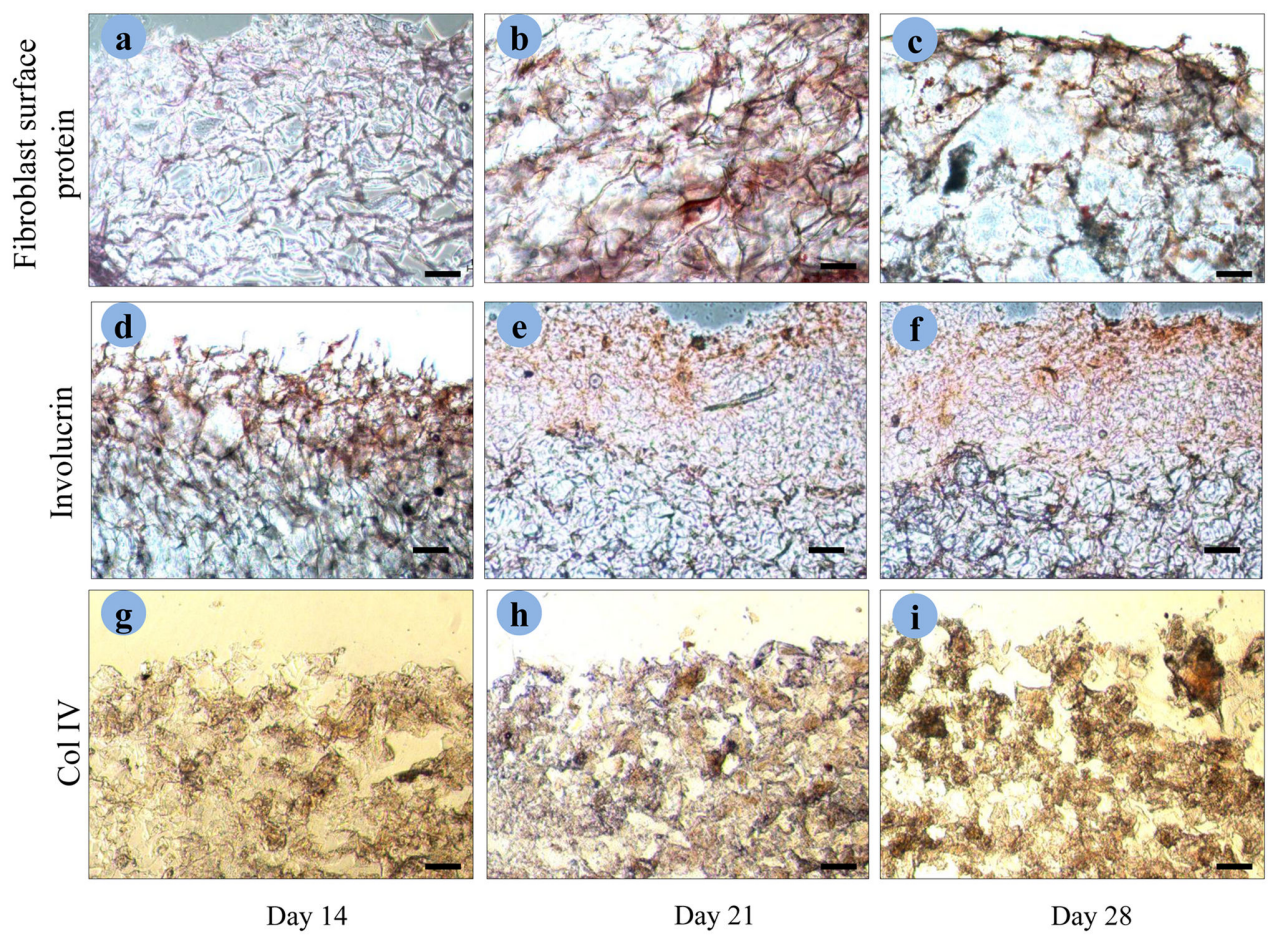
Expression of fibroblast surface protein, involucrin and collagen type IV in co-cultured sericin matrices. Immunohistochemical staining performed on deparaffinized sections using the HRP-conjugated secondary antibody and visualized using DAB as substrate. The antibodies used were anti-type IV collagen, mouse monoclonal anti-involucrin and mouse monoclonal anti fibroblast surface protein. Pictures were taken after 14, 21 and 28 days of culture at the air–liquid interface (scale bar = 100 µm).

The expression of differentiation markers in sericin co-culture model were studied by performing immunohistochemical studies on deparaffinized sections. Used antibodies were monoclonal involucrin antibody, monoclonal antibody to collagen type IV (Col IV) and monoclonal antibody to fibroblast surface protein. In sericin co-culture model, expression of fibroblast surface protein is present throughout the whole matrices section as shown in Figure 12 (a, b, c). The expression of involucrin is strongly observed as shown in Figure 12 (d,e, f). Positive staining for collagen IV (Col IV) is observed in later stage of growth Figure 12 (g, h, i).

### Cytokine production

**Figure 13:**
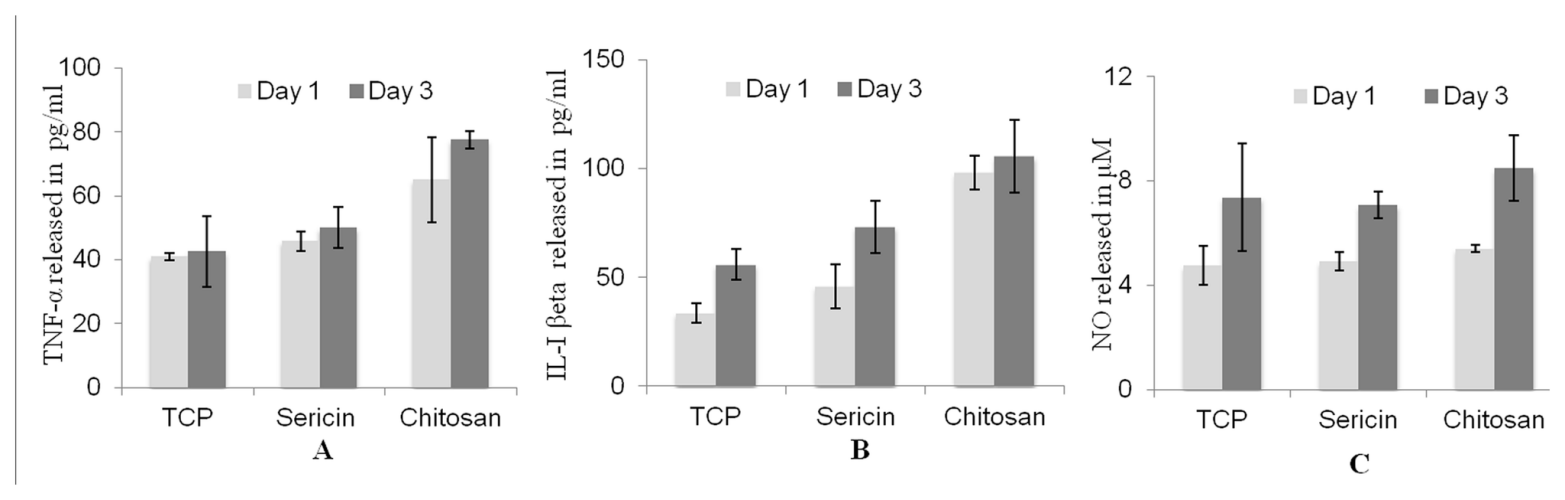
Production of TNF- α (A), IL-1β (B) and of nitric oxide (C) by RAW 264.7 murine macrophages on sericin and chitosan matrices with tissue culture plates (TCP as negative control) for day 1 and day 3. The TNF α, IL-1beta and nitric oxide production by the cells estimated from a standard graph.

TNF-α, IL-1β and NO production by RAW 264.7 macrophage for day 1 and day 3 are not significantly different from TCP as shown in Figure 13A, Figure 13B, and Figure 13C respectively.

## Discussion

Porous sericin matrices are fabricated using the freeze-drying protocol. Sericin is hydrophilic and amorphous in nature [20,26]. In the present work, the concentration of genipin and its crosslinking time with sericin matrices is constant. Genipin solution is prepared in PBS (pH 7.4), as it introduces nucleophilic agent that initiates the crosslinking reaction of genipin with itself, forming an oligomeric crosslinking agent [27,28]. This creates a higher degree of intermolecular binding, without causing residual toxicity to the cells.

The freeze dried technique creates regular pore sizes with well distribution of pore microstructure within the matrices. This provides an advantage to its application as tissue engineered matrices [29]. For tissue engineered matrices specifically for skin reconstitution porosity is a decisive factor and the pore structure of three-dimensional matrices influences cellular activity [30]. The porous nature of the matrices facilitates cell penetration and proliferation to a deeper level. This also ensures sufficient nutrient and oxygen transport that accelerates wound healing [31]. 3D porous matrices provides appropriate atmosphere suitable for cell proliferation and colonization. Further they also provide environment necessary for mass-transport of cell nutrition, cell migration and cell attachment. The pore size affects cell behaviour and also considerable change in cell behaviour occurs due to variation in pore sizes [32]. Too small pores restrict cell migration and reduce the distribution of nutrients and removal of waste products. Conversely, too large pores lack specific surface area that limits the beneficial effect of initial cell attachment [33]. Hence optimal pore size and specific surface area are important factor for migration and cell attachment. Tissues have several hierarchical configurations that differ from a length scale of 0.1-1 mm [34]. Cell-cell inter-relationships is control by subcellular structures in range of 1-10 µm and the essential functional units of the tissue is build up by supracellular scale structures exists in ranges of 100-1000 µm. The average sizes of pores created in sericin matrices are appropriate for fibroblasts and keratinocytes proliferation and rehabilitation in skin defects. Sericin being water soluble and amorphous protein has poor mechanical properties. The results indicate that the crosslinked sericin matrices have facilitated compact structure providing sufficient compressive properties hence mechanical stability to the sericin matrices. However the chitosan matrices are mechanically better than sericin matrices. Earlier reports show that sericin protein been chemical crosslinked and blended with other biopolymer/synthetic polymer to increase its mechanical properties in the form of films, gels, and scaffolds [20,22,23,35].

The swelling ability plays important role in tissue regeneration as it facilitates not only attachment but also promotes cells migration inside the matrices while the pore swells and increases in diameter [36]. This helps cells to grow in a three-dimension environment during *in vitro* culture. The swelling ratio of the matrices strongly depends on the hydrophilic nature and microstructure of the matrices [37]. As seen in the results the water retention ability of the hope sericin matrices decreases by the cross-linking and at the same time is lesser than chitosan matrices. SEM images clearly shows that the porosity of chitosan is more than sericin matrices hence it retains more solvent in it. Additionally chitosan possesses higher elasticity, which is helpful for the retention of the matrices original porous structure as well. The decrease swelling in genipin crosslinked sericin matrices may also be presumably related to the crosslinked sericin polypeptide. Generally cross-linking treatment reduces the hydrophilic groups like amino or carboxylic groups which get consumed during the cross-link reaction [37]. The degradation result indicates that after performing the cross-linking treatment with 0.5% w/v genipin solution for 24 h, the anti-degradation ability of sericin matrices enhanced.

*In vitro* cell proliferation and metabolic activity investigation performed by Alamar blue assay and live/dead assay on co-culture sericin and chitosan matrices demonstrates good cell proliferation and cell viability tendency. No significant changes are observed for the proliferation of co-cultured cells on sericin and chitosan matrices. The results indicate that both of these cell types have proliferative potential on sericin matrices as in chitosan matrices. Hydrophilic substrate is reported to promote interaction with negatively charged cell surface [38]. Sericin is hydrophilic in nature, possessing various biological properties, hence can promotes cell adhesion, spreading and proliferation.

Several cytokines play important role in wound healing and have been detected in skin as well as in keratinocyte and fibroblast cultures [10,39]. Keratinocytes stimulate fibroblasts to synthesize growth factors, which in turn stimulates keratinocytes proliferation in a double paracrine manner. Investigations have shown that keratinocytes interacts with fibroblasts in a TGF-beta dependent manner during the wound healing process [40]. Basic fibroblast growth factor in addition with VEGF induces angiogenesis. While IL-1, TNF, TGF-beta, b-FGF all work to increase the production of collagen locally. Keratinocytes strongly express TGF- beta1 and it modulates differentiation of fibroblasts to myofibroblasts which contribute to generation of a contractile force [41].

In co-cultured construct of sericin keratinocytes tends to cover the fibroblasts to form skin-like structures over a period of time. This reveals that keratinocytes and fibroblast is actively proliferating and direct contact of fibroblasts with keratinocytes stimulates the migration and proliferation of keratinocytes. One of a leading factor effecting re-epithelialization mechanism and re-establishment of tissue integrity is the cross talk between epidermal keratinocytes and fibroblasts [10]. Investigations have demonstrated that direct contact of fibroblasts with keratinocytes stimulates the migration and proliferation of keratinocytes during wound healing. Basically epithelialization is the migration, growth and differentiation of keratinocytes until epidermis forms and gets stratified [42]. Histological analysis of the co-culture on sericin matrices indicates regeneration of the dermis and the epidermis likewise in chitosan matrices. Stratification of the keratinocytes in sericin matrices within the epidermal layer resembles with the architecture similar to epithelial tissues generated with control chitosan matrices. This tissue architecture became multi-layered, well-differentiated and more prominent with time. The result suggests that co-culture of keratinocytes with fibroblast in sericin matrices provide evidence to be desirable matrices for skin tissue engineering.

Wound healing comprises complex mechanism involving inflammation, fibroplasia, neovascularisation, collagen deposition, epithelialization and wound contraction. Fibroblasts play important role in providing support framework to new blood vessel growth and epithelialization by directing collagen synthesis and deposition [3]. Most tissue ECM including the dermis of skin contains collagen. The predominant subtypes are type I and III with small amounts of type V and IV. The major component of the basement membrane is known to be type IV collagen. Type IV collagen, along with laminin, plays an important role in cell adhesion, migration, differentiation, and growth. The migration of human keratinocytes is an early and important event in the re-epithelialization of cutaneous wounds. Type IV collagen, a ubiquitous basement membrane component, promotes keratinocyte migration. In culture of keratinocytes on fibroblast-populated collagen matrix, laminin, and type IV and VII collagen were expressed more strongly than in the absence of fibroblasts [43]. Fibroblasts induced TGF-β secretion is shown to induce the synthesis of collagen type IV and VII by keratinocytes [44]. Expression of Col IV and fibroblast surface protein indicates increased concentrations of fibroblasts. Involucrin is a protein precursor of epidermal cornified layer and expressed specifically only in the upper layers in normal skin. Involucrin is a marker of keratinocytes differentiation. Involucrin on the upper layer of matrices indicates the significance of paracrine signaling between keratinocytes and fibroblasts. The characteristic distribution of involucrin, fibroblast surface protein, and expression of Col IV confirmed the capacity of co-culture cells to promote normal epithelial morphogenesis and differentiation in hope sericin matrices.

The sericin matrices do not induce any toxic effect. Implant material when come in contact with *in vivo* situation it comes in contact with inflammatory cells such as macrophages. Macrophage plays important role in normal wound healing and the reparative process. IL-1β and TNF α are pro-inflammatory cytokines and they acts synergistically during the initiation of the inflammation. Inflammation is the first response of the immune system mediated by inflammatory cells like macrophages to infection and is involved in maintenance of tissue homeostasis [45]. Chemical mediators are produced in response to an antigen that mediates many of the vascular and cellular responses of inﬂammation. Cytokines such as interleukin 1 (IL-1) and tumor necrosis factor (TNF) are example of chemical mediators of inﬂammation. Increase level of TNF-α result in programmed cell death (apoptosis) as well as enhance phagocytosis of neutrophils [46]. IL-1β is associated with tissue destruction by induction of tissue degrading proteinases [47]. Sericin is reported to have low inflammatory reactions *in vitro* and *in vivo* with wound healing process [24,20]. The results suggest that sericin matrices have no significant inflammatory response.

3D reconstructed skin has shown to express essential structural and functional components of native skin comprising ECM proteins and promotes cellular differentiation *in vivo* [48]. Previously 3D tissue engineered co-culture models has been reported for the *in vitro* testing of irritant chemicals at sub-toxic concentrations and for inflammatory and toxicity testing as an alternative to *in vivo* models [49,50]. The present work establishes sericin from “Sericin Hope” silkworm cocoons as a new material for dermal equivalent with enhanced biostability and good biocompatibility. The results indicate that the *in vitro* co-culture on hope sericin matrices with fibroblast-keratinocyte forms epidermal and dermal components on the matrice surface. The novelty of this work comes from (1) utilizing the natural silk protein sericin of high purity in large quantity and by means of less hydrolysis; (2) 3D keratinocytes-fibroblasts co-culture model is being developed as an alternative for *in vitro* skin replacements graft. The findings exhibit that our model may be useful as a skin equivalent for grafting.

## Materials and Methods

“Sericin Hope” cocoons were kindly obtained from Zhejiang Sci-Tech University, Zhejiang Province, China. Genipin, Sigma (St. Louis, USA), Fetal calf serum, trypsin–EDTA and penicillin–streptomycin antibiotics (Gibco BRL, USA), paraformaldehyde (Merck, India), Live/dead viability/cytotoxicity kit, ELISA kit for TNF-α, IL-β quantification (Invitrogen, USA), Cell Tracker red and green (Molecular Probes) were purchased for this study. All other chemicals used were of analytical grade.

### Preparation of Porous Matrices

Porous matrices of sericin obtained from “Sericin Hope” cocoons were prepared by freeze dried technique. Briefly, cut cocoon pieces were soaked in deionized water and autoclaved at 120 °C for 20 min. The solution obtained was centrifuged at 5000 rpm for 10 min to remove the insoluble material. The supernatant was cooled at room temperature, collected and concentrated against 30% PEG 6000 solution to get 2% w/v solution. The solution was poured into 24 (13 mm x 10 mm) or 96 (6 mm x 2 mm) well polystyrene culture dishes and frozen at −20 °C. The frozen samples were freeze-dried and treated with 70% ethanol to induce crystallinity. They were cross-linked with 0.5% w/v genipin solution in PBS (pH 7.4) for 24 h at room temperature. Afterwards the matrices were rinsed thoroughly in distilled water. Ethanol (70% v/v) sterilized samples were used for cell culture experiments.

In most of the experiments chitosan matrices were taken as control. For preparation of chitosan matrices, dried chitosan powder (Sigma, USA) was dissolved in 0.2 M acetic acid solution to prepare a 2% (w/v) solution. The chitosan matrices were prepared by pouring the solution into the 24 or 96 well plates, freezed at -20 °C and lyophilized for 10 h. To remove the residual acetic acid, the fabricated chitosan matrices were immersed in gradient ethanol solution ranging from 100% to 0% (100, 80, 50, 30, and 0%) for 2 h in each concentration. Finally, the chitosan matrices obtained were washed with deionized water and sterilized for cell culture experiments.

### Scanning electron microscopy (SEM)

The surface morphologies of the porous matrices (sericin and chitosan) were examined by SEM (JEOL, JSM-5800). The SEM images were acquired after gold sputtering at operating voltage of 20 kV. ImageJ 1.40g software was used to determine pore sizes (average of 30 random pores) within the porous matrices.

### Fourier transform infrared spectroscopy (FTIR) measurement

All infrared spectra were recorded in the range of 400–4000 cm^−1^ using Thermo Nicolet Magna 860 FTIR spectrometer. Each spectrum was acquired by accumulation of 32 scans with a resolution of 4 cm^-1^.

### Mechanical properties

The compressive mechanical properties of matrices (13 mm x 10 mm) were tested using a Universal Testing machine, Housfield-H25KS equipped with a 0.1 kN load cell at ambient room temperature using modification of ASTM method F451-95. The samples were pre-soaked in PBS for 2 h and were examined with crosshead speed of 1 mm/min. At least three specimens were tested for each sample.

### Swelling behaviour

Swelling properties were studied using conventional gravimetric procedure. The swelling behaviour of dried matrices were determined by immersing completely the dried matrices samples in phosphate buffered saline (PBS) solution (pH = 7.4, 3 mM NaN_3_) at 37 ± 1 °C for 24 h. The swelling ratio (SR) was calculated by the equation:

$$SR=\frac{Ww-Wd}{Wd}$$ (1)

Where *Ww* and *Wd* are the weights of the matrices at the swelling state and at the dry state, respectively.

### Proteolytic and hydrolytic degradations

Sericin matrices crosslinked and non-crosslinked samples were immersed in lysozyme 2U/ml solution for the proteolytic degradation study and in PBS (pH 7.4) for hydrolytic degradation study. The samples and solutions were kept at 37 ± 1 °C for the duration of the study and had the enzyme solution and PBS solutions changed regularly. For each mass measurement, the samples were removed from the respective solutions, dried and then weighed. Degradation profiles of the matrices were plotted for different time periods by calculating the percentage weight loss (WL) determined by the formula:

$$WL\left(\%\right)=\frac{\left(Wo-Wt\right)}{Wo}\times 100$$ (2)

Where W_0_ is the initial dry weight of the matrices and W_t_ is the final dry weight after duration of incubation.

### Maintenance of cell lines

Human dermal fibroblast cells were obtained from PromoCells (Heidelberg, Germany) and established cell line human keratinocyte (HaCaT) cells were obtained from animal cell culture repository National Centre for Cell Science (NCCS), Pune, India. Fibroblasts and keratinocytes were cultured in complete DMEM and in DMEM: DMEM F12 (3:1 ratio) medium respectively supplemented with 10% fetal bovine serum (FBS), 200 mm l-glutamine, 2 mg/ml sodium bicarbonate, and 100 µg/ml penicillin/streptomycin. The cells were cultured in tissue culture flasks at 37 °C in a humidified atmosphere of 5% CO_2_. Once the cells reached 80–90% confluent state, they were split using 0.05% trypsin/EDTA solution. The medium was replaced after every three days.

### Co-culture on fabricated matrices

Human dermal fibroblast cells and human keratinocyte (HaCaT) cells were cultured in media and trypsinised when reached 80–90% confluent state (Figure 1B (a and b)). Fibroblasts-keratinocytes co-culture was done on sericin constructs by seeding 1 × 10^5^/matrice (6 mm x 2mm) fibroblasts on sericin matrices pre-soaked with DMEM medium at day 0. On day 2, keratinocytes (1 × 10^6^) were directly seeded onto the surface of sericin matrices containing fibroblasts to create co-culture model. The construct were cultured further for 7 days in DMEM: DMEM F12 (3:1) medium supplemented with 10% FBS. After 7 days the co-culture matrices were raised to the air-liquid (A/L) interface to allow stratification of the keratinocytes on the matrices. All co-culture constructs were maintained at 37 °C with 5% CO_2_ and 100% relative humidity. Similarly fibroblasts-keratinocytes co-culture was done on chitosan matrices as control. Schematic representation of matrices preparation and fibroblasts-keratinocytes co-culture on construct is shown in Figure 1A.

### Alamar blue proliferation assay

Alamar blue assay was performed to assess the cell proliferation on the sericin matrices at different time periods. The Alamar blue assay measures quantitatively cell proliferation as well as relative cytotoxicity. It incorporates a water soluble colorimetric oxidation-reduction (Redox) indicator that changes colour in response to the chemical reduction of the culture medium resulting from cell growth (metabolic activity). Alamar blue dye (Invitrogen, USA) at a concentration of 10% v/v in PBS was added to each test well and incubated for 4 h at 37 °C in 5% CO_2_. 100 µl of the medium was transfered to a fresh 96 well plate and spectrophotometrically absorbance was read at 570 nm (reduction) and 600 nm (oxidation). The data were expressed as a fraction of the cell activity.

### Live/dead staining

The viability of cells seeded on sericin and chitosan matrices were investigated using the live/dead viability cytotoxicity kit (Invitrogen, USA) following the manufacturer’s protocol. At desired interval of time the seeded matrices were washed with PBS and incubated in 40 nM calcein AM (staining live cells green), 20 nM ethidium homodimer (EthD-1, staining dead cells red) in PBS for 30 min at 37 °C in the dark. The cross-section of the different matrices were imaged by confocal microscopy FV 1000 Advance software v. 4.1 (Olympus) with 488 nm and 534 nm excitation. Still images at various depths were captured and a series of micrographs were later combined for “z-stacked” compilation images.

### Confocal laser microscopy for cell proliferation

Proliferation of fibroblasts and keratinocytes on sericin and chitosan matrices was assessed by live cell imaging using confocal microscopy. For staining the cells, confluent flask of fibroblast and keratinocyte cells were trypsinised and centrifuged to obtain a cell pellet and supernatant was aspirated. Cells were resuspended gently in pre warmed (37 °C) medium containing submicromolar concentrations of cellTracker (Molecular Probes). To distinguish keratinocytes from fibroblasts in the co-culture, keratinocytes were stained with cellTracker red and fibroblasts were stained with cellTracker green. The fibroblasts (1 ×10^5^/ matrice) (6 mm x 2mm), were cultured on sericin matrices for 48 hours and then keratinocytes (1×10^6^/ matrice), were seeded onto the above fibroblasts seeded matrices. After specified time period the co-culture constructs were fixed in 4% paraformaldehyde for 15 min before the samples were imaged using confocal laser scanning microscope (CLSM, Olympus FV 1000 attached with inverted microscope IX 81, Japan) equipped with Argon (488 nm) and He Ne (534 nm) lasers.

### Cell attachment on sericin hope matrices

For cell attachment study fibroblasts and keratinocytes cell were seeded on sericin hope matrices at a density of 1 x 10^4^ cells/ matrice (6 mm x 2mm) and incubated in a 5% CO_2_ incubator for 72 h. After the pre-specified incubation period, the matrices were washed twice with PBS before fixing with 3% glutaraldehyde for at least 30 min at room temperature. One batch of the seeded scaffolds were then subjected to step dehydration with serial ethanol for 10 min each and coated with gold, for imaging using a scanning electron microscope (SEM) (JEOL, Model 5600LV). Another batch was stained with Rodhamine-phalloidin for actin and Hoechst for nucleus and observed under confocal microscope (Olympus).

### TGF β, b-FGF and IL-8 quantification by ELISA

Selected cytokines were quantified by enzyme-linked immunosorbent assays (ELISA) in aliquots of culture medium of co-cultures, fibroblast with initial seeding of (1 ×10^5^), and keratinocytes with initial seeding of (1×10^6^) monoculture on sericin matrices after 72 hours. ELISA kits for IL-8 was purchased from R&D Systems (Wiesbaden, Germany), TGF-β (sigma, USA), and for b-FGF from R&D Systems (Wiesbaden, Germany). Protein values were calculated as pg/10^5^ cells and mean values ± standard deviation of data derived from duplicate measurements from 2 independent experiments are given.

### H&E staining

For histology, specimens of co-culture on sericin and chitosan matrices were fixed in 4% formaldehyde (in PBS, pH 7.4) and then embedded with paraffin. The 10 µm thick paraffin sections were stained with hematoxylin-eosin (HE) reagent for histological observations.

### Immunohistochemistry of matrice sections

After 7, 14 and 21 days of cultures at the air–liquid interface, matrices were fixed and embedded in paraffin. Sections of 10 µm of the matrices were, incubated for 12 h at 4°C with mouse anti-bovine type IV collagen IgG (Col IV), mouse monoclonal anti-involucrin and mouse monoclonal anti fibroblast surface protein IgG (Sigma-Aldrich), (diluted 1:100) and washed with PBS (pH 7.4) (3 times, each for 5 min). Subsequently, the sections were incubated for 30 min at 37 °C with HRP conjugated secondary antibodies followed by the chromogen detection using DAB as substrate.

### Macrophage stimulation assay

The immunogenicity of the sericin and chitosan matrices were evaluated by *in vitro* quantitative determination of TNF-α, IL-1β and nitric oxide concentrations in cell culture supernatant. Briefly macrophages (10^5^ cells/ construct) were seeded on sericin and chitosan matrices. Complete media was replaced with incomplete media after 24 h of cell seeding. Cell supernatant was collected and quantified for TNF-α and IL-1β level using ELISA kit (Invitrogen) after 24 and 72 h. Data accumulation was done at 450 nm and 600 nm using a microplate reader. To quantify the NO production cell supernatant were collected and incubated with Griess Reagent (Sigma) for 10 min. Data accumulation was done at 548 nm and 600 nm using microplate reader.

### Statistical analysis

Statistical analysis of the data was performed by one-way analysis of variance (ANOVA). Differences between experimental groups at a level of P ≤ 0.05 were considered as statistically significant and those at P ≤ 0.01 as highly significant.

## Conclusion

This study indicates the good growth of co-cultured fibroblasts and keratinocytes *in vitro* on 3D sericin matrices. This co-cultured construct could be delivered to a wound bed and may provide the wound closure and eventually accelerate the healing process. The 3D tissue-engineered skin model is a potential source of living skin equivalents for grafting *in vivo*. Further work should seek to evaluate how this functional construct behaves *in vivo* when seeded with autologous fibroblasts and keratinocytes. Follow-up study is needed to evaluate its fate after grafting more precisely.
